# Adopt, ignore, or kill? Male poison frogs adjust parental decisions according to their territorial status

**DOI:** 10.1038/srep43544

**Published:** 2017-03-06

**Authors:** Eva Ringler, Kristina Barbara Beck, Steffen Weinlein, Ludwig Huber, Max Ringler

**Affiliations:** 1 Messerli Research Institute, University of Veterinary Medicine Vienna, Medical University Vienna, University of Vienna, Veterinaerplatz 1, A- 1210 Vienna, Austria; 2 University of Vienna, Department of Integrative Zoology, Althanstrasse 14, A-1090 Vienna, Austria; 3 University of California Los Angeles, Department of Ecology and Evolutionary Biology, 621 Charles E. Young Drive South, CA 90095, Los Angeles, USA

## Abstract

Systematic infanticide of unrelated young has been reported in several animal taxa. Particular attention has been given to carnivores and primates, where infanticide is a sexually selected strategy of males to gain increased access to female mating partners. Cannibals must ensure avoiding their own offspring and targeting only unrelated young. Therefore, decision rules are needed to mediate parental and cannibalistic behaviour. Here we show experimentally that male poison frogs adjust their parental responses – care or infanticide – towards unrelated clutches according to their territorial status. Male frogs followed the simple rule ‘care for any clutch’ inside their territory, but immediately switched to cannibalism when establishing a new territory. This demonstrates that simple cognitive rules can mediate complex behaviours such as parental care, and that care and cannibalism are antagonistically linked. Non-parental infanticide is mediated by territorial cues and presumably serves to prevent misdirected care in this poison frog. Our results thus prompt a re-consideration of evolutionary and causal aspects of parental decision making, by suggesting that selective infanticide of unrelated young may generally become adaptive when the risks and costs of misdirected care are high.

Supportive behaviour towards one’s own offspring can increase the parent’s fitness directly, while detrimental behaviour towards unrelated progeny can increase individual fitness relative to others^1^. parents are expected to employ behavioural strategies that minimize the errors of accidentally adopting unrelated offspring or penalizing one’s own offspring, by following reliable decision rules across varying social, temporal and spatial contexts^2^. In order to establish adaptive decision rules in parenting, individuals must be able to accurately evaluate the reproductive value of all behavioural options^3^ and, if circumstances change and new information becomes available, perform a behavioural switch against the previous predisposition^2^,^4^. When such behavioural flexibility occurs in so-called ‘higher’ vertebrates, such as mammals and birds, this is often interpreted as being the outcome of general intelligence, which includes reasoning, planning, and abstract thinking^5^,^6^,^7^. However, recent studies have shown that also simple decision rules can solve complex problems across various contexts, including parental care^8^,^9^,^10^.

Both the care for non-filial offspring (‘alloparenting’) as well as their systematic, rule-based killing (‘non-parental infanticide’) are reported frequently from behavioural observations on group-living mammals and birds^11^,^12^,^13^. In most of these taxa, killing other individuals’ young is described as a sexually selected strategy by males to obtain increased spatial resources, social status or attractiveness, and to make females become receptive earlier by terminating their nursing duties^12^,^14^,^15^. In turn, the perpetrator may obtain nutritional gains or reduce resource competition for its own offspring by eliminating other parents’ young^12^,^15^. Nest-adoption as well as egg cannibalism are also known from several arthropods and fish^16^,^17^,^18^,^19^,^20^,^21^. In these species, egg cannibalism is a common strategy to offset reduced foraging during parental duties^21^,^22^, or to prevent the spread of microbial infections by moulded eggs^23^. In harvestmen and spiders, cannibalism by conspecific males and females might even be the main source of clutch mortality^17^,^24^,^25^, motivated by the nutritional value of a clutch^19^. The adoption of unrelated eggs (or active nest take overs) by males is mainly observed in species where females prefer males that already guard clutches^17^,^26^, indicating that elevated mating opportunities might outweigh increased parental efforts.

Currently, there is only sparse evidence that infanticide may also insure against misdirected parental care, the accidental adoption of unrelated offspring^27^. Few examples come from group-living mammals, where the aggressive/infanticidal behaviour serves to prevent milk theft (e.g. female pinnipeds^28^, female black-tailed prairie dogs^29^), or to avoid alloparental over-winter care (e.g. male golden marmots^30^). Further support comes from some species of fish^21^,^31^,^32^ and harvestmen^24^. These examples suggest that selective infanticide of unrelated young may generally become adaptive when the risk and costs of misdirected care are high. The exact cues used for assessing putative parentage are often obscured under natural conditions. Therefore, controlled manipulation experiments can help to elucidate how parents adjust their parental decisions.

In Neotropical poison frogs (Dendrobatidae), parental care by one or both sexes is considered a synapomorphy of the entire family, with the ancestral state being paternal care, comprising egg attendance and transport of the tadpoles to aquatic sites for final development^33^,^34^,^35^,^36^,^37^. Territoriality and site fidelity of one or both sexes are also common features of the family and found in most species^36^,^38^. In *Allobates femoralis* (Dendrobatidae, Aromobatinae) males are highly territorial and show site fidelity throughout the breeding season^39^,^40^. The prominent male advertisement call serves to attract female mating partners as well as to mediate spacing between males; however, conspecific male intruders are physically attacked by territory holders^41^. Females occupy perches interspersed between male territories, but do not show any aggressive interactions to either sex^40^,^42^. Both sexes are iteroparous and highly polygamous throughout the reproductive season^43^. Courtship, mating, and terrestrial oviposition take place in the leaf litter inside the male’s territory^42^,^44^, therefore possessing a territory is a prerequisite for male reproductive success^43^. After three weeks of larval development in the clutch the male transports the hatched tadpoles to water bodies that are usually outside a male’s territory^45^,^46^, with males distributing tadpoles across several sites for bet-hedging^47^. A recent study showed that males also transport unrelated clutches that are placed inside their territory^48^, indicating a strong predisposition of territorial males to perform tadpole transport. Although females abandon their clutches and return to their perches immediately after oviposition, they do take over tadpole transport when the father disappears before tadpole transport is due^49^, identifying their own clutches solely based on location^48^. However, contrasting the previous assumption of a strong motivation to transport any conspecific tadpoles, we observed a case of clutch cannibalism by an adult male during a territory takeover during an ongoing follow-up experiment on male removal in the field (Weinlein, pers. obs.; see movie S1). Both alloparental care and clutch cannibalism have previously been reported in dendrobatid frogs in captivity^50^,^51^ and in the field^52^, but the factors controlling adaptive decision making in poison frog parental care remain unknown. We therefore designed an experiment to investigate whether male infanticide is mediated by territorial cues in *A. femoralis*.

Our study design is based on the manipulation of the territorial status of males (Fig. 1). In the ‘takeover’ group, we simulated a territory takeover by transferring males (*N* = 10) from their home terraria to novel, empty terraria. In the ‘resident’ group, we captured males (*N* = 10) and returned them to their home terraria after the same handling procedure used in the first experiment. In all trials, we placed unrelated clutches of conspecific parents inside the terraria before releasing the focal males. All clutches we used contained embryos that were already visibly elongated (9 to 31 days after oviposition) without a significant difference between experimental groups. All trials were filmed to record the occurrence and/or frequency of cannibalism and tadpole transport. Supplementary text, movies (S1 to S4), and raw data can be found in the supplementary materials.

## Results

**Figure 2:**
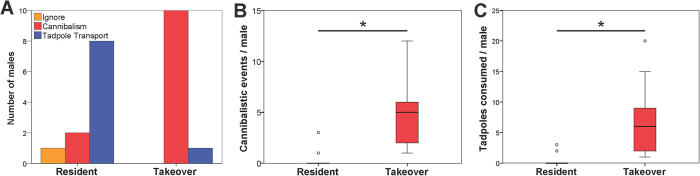
Male responses across the two experimental conditions. Male behaviour towards unrelated clutches was strongly context dependent: (**A**) ‘Residents’ mainly responded with parental care, while all ‘takeover’ males preyed on the clutches; (**B**) the frequency of cannibalistic events and (**C**) the total number of tadpoles consumed were significantly higher in ‘takeover’ males.

Significantly more ‘takeover’ (t) males than ‘residents’ (r) were cannibalistic (10 of 10 vs. 2 of 10 males, Fisher Exact test, *P* < 0.001, Fig. 2A), they fed more often (median_t/r_ = 5/0; Mann-Whitney *U*-test, *U* = 4.5, *P* < 0.001, Fig. 2B), and consumed more tadpoles (median_t/r_ = 6/0; Mann-Whitney *U*-test, *U* = 5.5, *P* < 0.001, Fig. 2C). In contrast, ‘takeover’ males transported tadpoles significantly less often than ‘residents’ (8 of 10 vs. 1 of 10 males; Fisher Exact test, *P* = 0.0055, Fig. 2A). Additional notes on behavioural differences between the two experimental groups (snapping, moistening) are given in the supplementary text, and are in line with the results above in demonstrating different parental decision rules, according to territory status.

## Discussion

In *A. femoralis* males, parental decisions are mediated by territorial cues. Territory holders follow the simple decision rule ‘care for any clutch inside my territory’, but immediately switch to cannibalism when taking over a new territory. We suggest this context-dependent behavioural switch between infanticide and parental care is adaptive as it effectively allocates parental effort while minimizing the risk for parental errors at minimum cognitive effort. It also acts as an antagonistic backup strategy to the previously demonstrated high predisposition of *A. femoralis* males for indiscriminate tadpole transport^48^ by preventing takeover males from transporting clutches of the former territory holder. Similar behaviour was reported for a harvestman^24^ and recently also for the plainfin midshipman fish, where males reduce their parental investment and/or cannibalize foreign eggs after nest takeover^20^,^31^. In *A. femoralis* the likelihood for males to encounter foreign clutches inside their own, established territory can be considered rather low as territories are vigorously defended physically against male intruders^41^. This notion is further supported by a previous study which reported only four (3%) out of 119 tadpole transporting males carrying unrelated tadpoles (in three cases the tadpoles of neighbouring individuals) on their back^45^. By following the simple decision rule ‘transport all clutches inside and eat all clutches outside my territory’, male *A. femoralis* reduce both the risk of accidentally rejecting own offspring at the time a territory has been established as well as the risk of misdirected care at locations where the likelihood of paternity is low. Cannibalistic males likely gain additional nutritional benefits, as amphibian larvae constitute high quality food^53^. We can exclude purely nutritional motivation for the observed infanticide, as the feeding regime was constantly high and equal for both experimental groups, and did not differ from pre- and post-experimental conditions. Therefore, we suggest that similar behavioural strategies are likely to have evolved in other territorial and/or nest-brooding species that are at risk of parental exploitation, particularly under circumstances when the respective likelihood of encountering unrelated and own offspring is predictable, for species with brood parasitism, or when takeovers of inhabited breeding sites happen regularly.

However, the adoption of unrelated clutches or offspring is not necessarily associated with high costs. In several arthropods and fish, for example, females prefer male partners that already guard clutches^17^,^26^. In these species, adoption and takeover of foreign clutches by males are common, suggesting that the increased likelihood for obtaining mating partners outweighs the elevated costs of parental care. Apparently, this is not the case in *A. femoralis*, where clutch adoption happens rarely under natural conditions, and males likely do not attempt to increase their attractiveness this way. It rather seems that reduced misdirected care and nutritional benefits from cannibalism outweigh the potential benefits of adoption, and thereby promoted the evolution and maintenance of infanticidal behaviour after territory takeover.

As selective non-parental infanticide is well known from group-living mammals and birds, this previously lead to a focus on social parameters (e.g. breeder-helper status, dominance rank, group size) when thinking about adaptive benefits of this behaviour^13^. As highly social species are considered to feature increased cognitive abilities and learning capacities^54^ (but see ref. 55), this might mislead to simply attribute flexible parental decision making to certain cognitive skills. However, parental behaviour is observed in a wide range of taxa with varying levels of cognitive complexity^56^,^57^, suggesting that also simple decision rules can generate complex and context-dependent behavioural responses and provide convergent solutions to similar challenges. Our findings demonstrate that parental decision making, involving a flexible switch between care and infanticide to avoid misdirected care, can evolve in the absence of group-living and in species with a comparatively simple brain organization^9^,^10^,^58^. By applying this simple decision rule, males can effectively allocate parental effort and perform non-parental infanticide without more sophisticated offspring recognition mechanisms.

Across the animal kingdom, various offspring discrimination strategies have evolved that mediate parental decision making^59^. Commonly, the cues assessed for evaluating putative parentage are linked directly to the identity of the progeny – or contextual cues directly linked to the reproductive event, such as timing since mating, spatial location of offspring, or offspring age^2^. In fish, clutches are commonly cannibalized if the certainty of parentage for the caring parent is low (e.g. due to sneaker interferance^60^ or by assessing direct offspring cues^32^,^61^). This reflects the trade-off between (low) current reproductive success and the production and care for future broods^62^. However, cues that are only indirectly linked with the reproductive event may also be used for predicting parentage. In burying beetles, for example, both males and females show time-dependent shifts from infanticide to parental behaviour according to the light period after carcass discovery^63^. In our study, males adjusted their parental decisions according to their territorial status, thereby using a proxy for the likelihood of encountering related young that is decoupled from the actual reproductive event. And although such behaviour potentially allows for active exploitation by other pairs (i.e. cuckoo’s behaviour), this is presumably precluded by the high territoriality and elaborate courtship behaviour of *A. femoralis*, which is also a general characteristic of dendrobatid frogs^36^. For the two ‘residents’ that preyed on clutches inside their terrarium, we cannot rule out the possibility that some males can identify unrelated clutches, or that they misinterpreted their territorial status after the control-handling. Further studies using controlled manipulation experiments are needed to uncover the cues by which parents adjust their parental decisions. Comparisons between diverse animal taxa, with different social organisation and parental systems will help us understand how different parental strategies are promoted and maintained over evolutionary time.

Our study further suggests that parental care and non-parental infanticide are antagonistically linked; see also ref. 64 for studies in mice. Recent research has emphasized that feeding and parental behaviours might be regulated antagonistically via a common physiological pathway^65^. Together with our findings this calls for further integrative investigations on the interplay between neuronal and hormonal activity in territoriality, mating, feeding, and parental care^66^.

## Methods

### Laboratory frog population

This study was conducted from 17 December 2015 to 17 February 2016 in the animal care facilities at the University of Vienna. All frogs were housed in standard glass terraria of equal size (60 × 40 cm and 40 cm high) with identical equipment and furnishing. The floor was covered with pebbles of expanded clay, the back and side walls are covered with xaxim (plates made of dried tree fern stems) and cork mats, and the front was covered with fabric to prevent visual contact between neighbouring terraria and disturbances during maintenance. All terraria contained half a coconut shell, a small plant and a branch as suitable shelters and calling positions. We provided oak leaves as a substrate for oviposition, and a small glass bowl of 12 cm diameter filled with approximately 350 ml of water for tadpole deposition. An automatic raining, heating and lighting system ensured standardized climatic conditions with similar parameters to the natural conditions in French Guiana (temperature cycle between 19 °C (night) and 30 °C (day), 100% humidity, light from 7 a.m. to 7 p.m.) in all terraria. All frogs were fed with wingless fruit flies every second day.

### Ethical note

All frogs that were used in this study are part of an *ex situ* laboratory population at the animal care facilities at the University of Vienna. Permissions for sampling and export of wild-caught frogs were obtained from the responsible French authorities (DIREN: Arrete n° 82 du 10.08.2012 and Arrete n° 4 du 14.01.2013). All experimental procedures were discussed and approved by the ethics animal welfare committees of the University of Vienna and of the University of Veterinary Medicine Vienna in accordance with good scientific practice guidelines and current Austrian legislation. We followed the ASAB guidelines for the treatment of animals in behavioural research and teaching.

### Experimental design

All tested males (*N* = 20) were adult and had already sired clutches previously. We only tested males that were actively calling, thus showing territorial behaviour, at the onset of the experiment. Prior to testing, all males were kept isolated for about two weeks after removing the previous partner or after any remaining clutches were transported. Males were indiscriminately assigned to either the ‘takeover’ (*N* = 10) or the ‘resident’ (*N* = 10) group. In the ‘takeover’ group, all males were removed from their original terraria and transferred to another empty terrarium to simulate a territory takeover. In the ‘resident’ group, all individuals were caught but then returned to their own terrarium to control for eventual handling effects. In both groups, unrelated clutches of other breeding pairs were placed inside the terraria before males were released. We only used clutches containing tadpoles that had reached at least Gosner stage 17^67^, to avoid using clutches that fail to develop in the course of the experiment due to developmental issues. We tried to match the number of tadpoles per clutch and the respective developmental stage (i.e. days after oviposition) across groups, which were not significantly different between ‘resident’ and ‘takeover’ males (Student’s *t*-test; clutch size: mean_takeover_ = 15.8; mean_resident_ = 12.9; *t* = −1.345, df = 12.635, *P* = 0.202; days after oviposition: mean_takeover_ = 15; mean_resident_ = 17.3; *t* = 0.867, df = 18, *P* = 0.398).

### Data collection and analysis

A high-resolution video surveillance system consisting of 12 digital full-HD video surveillance cameras (IndigoVision, BX400 HD Minidome) was used for visual monitoring of the behavioural actions of individual frogs. Cameras were placed on top of the terraria and adjusted so that the entire clutch could be observed. Filming took place between 7 am and 7 pm, according to the automatic day-night light cycle of the entire room. Clutches were also visually inspected every other day to verify the present number of tadpoles within clutches and eventual tadpole transport by *A. femoralis* males, as assessed from video recordings.

Based on the video recordings we registered the following behaviours for all trials (1) presence/absence of cannibalistic behaviour; (2) number of cannibalistic events, (3) total number of tadpoles consumed per trial; (4) presence/absence of snapping behaviour towards the clutch; (5) number of snapping events, (6) presence/absence of deposition of tadpoles into the provided water bowl. Snapping refers to all snapping movements towards the clutch that do not result in consumption of any tadpole. Additionally, we also recorded if individuals were actively moistening the clutch. The ratios of behavioural responses were compared between test groups using Fisher Exact tests and Mann-Whitney *U*-tests where applicable. All statistical analyses were performed in IBM SPSS Statistics 23. Alpha for rejection of null hypotheses was set a priori at *P* < 0.05.

## Additional Information

**How to cite this article:** Ringler, E. *et al*. Adopt, ignore, or kill? Male poison frogs adjust parental decisions according to their territorial status. *Sci. Rep.* **7**, 43544; doi: 10.1038/srep43544 (2017).

**Publisher's note:** Springer Nature remains neutral with regard to jurisdictional claims in published maps and institutional affiliations.

## Supplementary Material

Supplementary Movie S1

Supplementary Movie S2

Supplementary Movie S3

Supplementary Movie S4

Supplementary Information

## Figures and Tables

**Figure 1:**
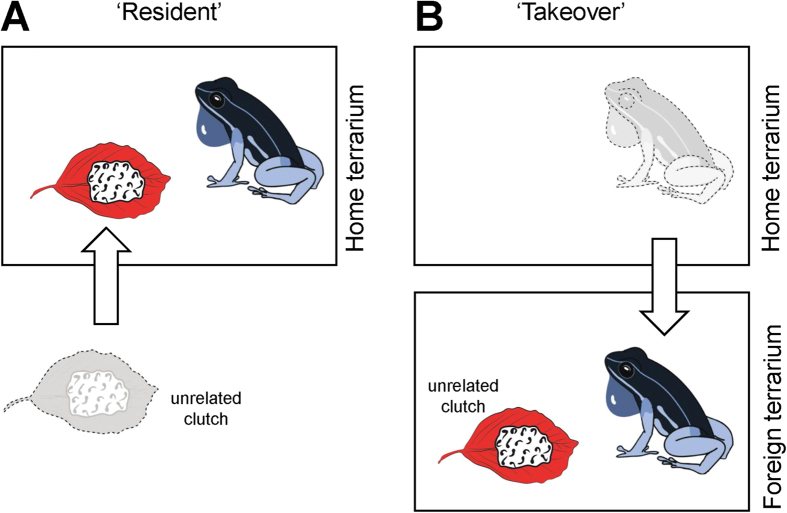
Experimental design. After capture, (**A**) half of the males were returned to their own terrarium (“Resident”), (**B**) the other half was transferred to a new terrarium (“Takeover”). In both experimental treatments an unrelated clutch was placed inside the tank before males were released. Picture drawn by Nadja Kavcik-Graumann and Andrius Pašukonis.
