# Supplementary material for: Adopt, ignore, or kill? Male poison frogs adjust parental decisions according to their territorial status
Source: Sci Rep. 2017 Mar 6;7:43544. doi: 10.1038/srep43544 (PMC5337939; doi:10.1038/srep43544)
Supplement: Supplementary Information [file srep43544-s5.pdf]

Supplementary Materials for

Adopt, ignore, or kill? Male poison frogs adjust parental decisions according to their territorial status

Eva Ringler, Kristina Barbara Beck, Steffen Weinlein, Ludwig Huber, Max Ringler
correspondence to: eva.ringler@vetmeduni.ac.at

This PDF file includes:

Supplementary Text
Captions for movies S1 to S4
Captions for databases S1 to S2
Databases S1 to S2

Other Supplementary Materials for this manuscript includes the following:

Movies S1 to S4

Supplementary Text

Cannibalism observed in the field

On 30 March 2014 a natural case of clutch cannibalism by a male *A. femoralis* was observed during the course of an ongoing male removal experiment (Weinlein, in prep). Ten days after the resident male was temporally removed from his territory, in order to elicit female tadpole transport (cf. ref 49), another neighbouring male entered the territory, fed on the removed male's clutch (see movie S1), and establish his new territory in this location during the following days.

Supplementary notes

Snapping: We recorded the presence and also frequency of snapping behaviour. Snapping occurred significantly more in 'takeover' than 'resident' males (10 of 10 versus 1 of 10 males, Fisher Exact test, $P < 0.001$), and also at higher frequencies (Mann-Whitney U-test, $U = 2.5$, $P < 0.001$). As these parameters were strongly linked with cannibalistic behaviours (i.e. all individuals that displayed snapping behaviour also preyed on the clutches), we did not further analyse snapping data.

Moistening: As this could only be observed unambiguously when leaves had dried up, which rarely happened due to the automatic raining system, moistening was not included in the comparative analyses. The only two cases where active clutch moistening could be reliably observed was in 'residents'.

Snapping and moistening were previously not reported from *A. femoralis*. Therefore, we added these anecdotal observations, as they might potentially be interesting for comparative work in other dendrobatid species in the future.

Movie S1

Clutch cannibalism by male *A. femoralis* in the wild. Initial observation in the field in French Guiana from 30 March 2014, where an adult male took over the territory of another male that was temporarily removed. The intruding male was observed via a surveillance camera to repeatedly prey on one clutch of the removed male. Meanwhile an ant passes by and also preys on one tadpole. The video has been cut to remove all stationary sequences; in real time.

Movie S2

Clutch cannibalism by male *A. femoralis* in the lab. One male of the 'takeover' group preys on the clutch. The video has been cut to remove all stationary sequences; in real time.

Movie S3

Clutch moistening by male *A. femoralis* in the lab. One 'resident' male actively moistens the clutch. The video has been cut to remove all stationary sequences; fast motion (x16).

Movie S4

Tadpole pick up and transport by male *A. femoralis* in the lab. 'Resident' male rotates on the clutch and tadpoles wiggle up his back; he even collects the last tadpole that initially did not manage to climb on his back. The video has been cut to remove all stationary sequences; fast motion (x8).

Database S1

List of all trials. R = Resident, T = Takeover, start = start of the trial, end = end of the trial, oviposition = date when the provided clutch was produced, dao = days after oviposition at the given start of the trial, n_tp = number of tadpoles within the clutch.

Test	Male	start	end	oviposition	dao	n_tp
R	m24	07.12.2015	02.01.2016	23.11.2015	14	19
R	m52	04.01.2016	17.01.2016	15.12.2015	20	9
R	m89	07.01.2016	18.01.2016	20.12.2015	18	9
R	m44	15.01.2016	25.01.2016	20.12.2015	26	12
R	m5	20.01.2016	10.02.2016	05.01.2016	15	14
R	m74	22.01.2016	28.01.2016	01.01.2016	21	13
R	m23	20.01.2016	01.02.2016	01.01.2016	19	14
R	m48	25.01.2016	10.02.2016	13.01.2016	12	14
R	m176	03.02.2016	12.02.2016	15.01.2016	19	12
R	m25	04.02.2016	17.02.2016	26.01.2016	9	13
T	m2	17.12.2015	02.01.2016	07.12.2015	10	19
T	m13	17.12.2015	22.12.2015	04.12.2015	13	7
T	m22	17.12.2015	06.01.2016	07.12.2015	10	23
T	m3	04.01.2016	14.01.2016	15.12.2015	20	26
T	m35	04.01.2016	15.01.2016	15.12.2015	20	16
T	m98	20.01.2016	03.02.2016	10.01.2016	10	8
T	m110	20.01.2016	28.01.2016	20.12.2015	31	13
T	m117	26.01.2016	04.02.2016	13.01.2016	13	13
T	m179	28.01.2016	16.02.2016	18.01.2016	10	20
T	m132	28.01.2016	07.02.2016	15.01.2016	13	13

Database S2

Behavioural responses of male *A. femoralis*. R = Resident, T = Takeover, cannibalism = presence/absence of cannibalism, n_cannibalism = number of cannibalistic events per trial, sum_tp = total number of tadpoles consumed per trial, snapping = presence/absence of snapping behaviour, n_snapping = number of snapping events, tt = presence/absence of tadpole transport.

Test	Male	cannibalism	n_cannibalism	sum_tp	snapping	n_snapping	tt
R	m24	0	0	0	0	0	0
R	m52	0	0	0	0	0	1
R	m89	0	0	0	0	0	1
R	m44	1	1	2	0	0	0
R	m5	0	0	0	0	0	1
R	m74	0	0	0	0	0	1
R	m23	0	0	0	0	0	1
R	m48	1	3	3	1	1	1
R	m176	0	0	0	0	0	1
R	m25	0	0	0	0	0	1
T	m2	1	6	9	1	2	0
T	m13	1	2	3	1	1	0
T	m22	1	12	20	1	7	0
T	m3	1	7	15	1	4	0
T	m35	1	5	6	1	1	0
T	m98	1	2	2	1	1	1
T	m110	1	2	2	1	3	0
T	m117	1	1	1	1	1	0
T	m179	1	6	8	1	1	0
T	m132	1	5	6	1	4	0
